# Assessment of hemodynamic efficacy and safety of 6% hydroxyethylstarch 130/0.4 vs. 0.9% NaCl fluid replacement in patients with severe sepsis: The CRYSTMAS study

**DOI:** 10.1186/cc11358

**Published:** 2012-05-24

**Authors:** Bertrand Guidet, Olivier Martinet, Thierry Boulain, Francois Philippart, Jean François Poussel, Julien Maizel, Xavier Forceville, Marc Feissel, Michel Hasselmann, Alexandra Heininger, Hugo Van Aken

**Affiliations:** 1 Réanimation médicale, Assistance Publique - Hôpitaux de Paris, Hôpital Saint-Antoine, 184 rue du Faubourg Saint Antoine, Paris, F-75012, France; 2 UPMC Université Paris 06, 4 Place Jussieu, Paris, 75005, France; 3 Inserm, Unité de Recherche en Épidémiologie Systèmes d'Information et Modélisation (U707), Paris, F-75012, France; 4 Réanimation Médicale, Hôpital Civil de Strasbourg, Place de l'Hôpital, 67091, Strasbourg, France; 5 Réanimation Polyvalente, Hôpital de la Source, 1Rue Porte Madeleine, Orléans, 45032, France; 6 Réanimation Polyvalente, Hôpital Saint-Joseph, 185 Rue Raymond Losserand, Paris, 75014, France; 7 Université Paris Descartes, 12 Rue de L'École de Médecine, Paris, 75006, France; 8 Réanimation Polyvalente, Centre Hospitalier de Metz, 1 Place Philippe de Vigneulles, Metz cedex, 57038, France; 9 Réanimation Médicale, Hôpital Sud, Avenue René Laënnec-Salouël, Amiens, 80054, France; 10 Réanimation, CH Meaux, 6-8 Rue Saint-Fiacre, Meaux, 77104, France; 11 Réanimation et Maladies Infectieuses, Site de Belfort, CH de Belfort-Montbeliard, 14 Rue de Mulhouse, Belfort, 90016, France; 12 Klinik für Anästhesiologie und Intensivmedizin, Universitätsklinikum, Hoppe-Seyler-Straße 3, Tübingen, 72076, Germany; 13 Klinik und Poliklinik für Anästhesiologie und Operative Intensivmedizin der Westf, Wilhelms-Universität, Albert-Schweitzer-Str. 33, Münster, 48149, Germany

## Abstract

**Introduction:**

Inadequate initial treatment and delayed hemodynamic stabilization (HDS) may be associated with increased risk of death in severe sepsis patients.

**Methods:**

In order to compare the hemodynamic efficacy and safety of 6% HES 130/0.4 and NaCl 0.9% for HDS in patients with severe sepsis, we designed a prospective, multicenter, active-controlled, double-blind, randomized study in intensive care units.

**Results:**

174 out of 196 patients reached HDS (88 and 86 patients for HES and NaCl, respectively). Significantly less HES was used to reach HDS vs. NaCl (1,379 ±886 ml in the HES group and 1,709 ±1,164 ml in the NaCl group (mean difference = -331± 1,033, 95% CI -640 to -21, *P *= 0.0185). Time to reach HDS was 11.8 10.1 hours vs. 14.3 ±11.1 hours for HES and NaCl, respectively. Total quantity of study drug infused over four consecutive days, ICU and hospital LOS, and area under the curve of SOFA score were comparable. Acute renal failure occurred in 24 (24.5%) and 19 (20%) patients for HES and NaCl, respectively (*P *= 0.454). There was no difference between AKIN and RIFLE criteria among groups and no difference in mortality, coagulation, or pruritus up to 90 days after treatment initiation.

**Conclusion:**

Significantly less volume was required to achieve HDS for HES vs. NaCl in the initial phase of fluid resuscitation in severe sepsis patients without any difference for adverse events in both groups.

**ClinicalTrials.gov:**

NCT00464204

## Introduction

Inadequate initial treatment and delayed hemodynamic stabilization (HDS) may be associated with increased risk of death in patients with severe sepsis [1]. Optimized management in the first 6 hours has been reported to significantly reduce mortality in patients with severe sepsis and septic shock [2]. Early adequate fluid resuscitation is a major step in the management of severe sepsis/shock and is recommended worldwide to improve prognosis [3]. Early restoration of intravascular volume requires aggressive filling with crystalloids or colloids.

A strategy that includes colloids that are able to reduce the amount of fluid required to reach HDS could be beneficial. However, there are concerns regarding the safety of colloids. Some data suggest hyperoncotic colloids and starches with a molar substitution >0.4 may be harmful for the kidney in patients with septic shock [4-6]. Third generation hydroxyethyl starch (HES), the so-called tetrastarch (molar degree of substitution 0.4 and medium molecular weight of 130 kDa) with a reportedly improved safety profile [7,8], has led to renewed interest in the use of HES for volume therapy.

However, no randomized controlled trial of tetrastarch in patients with sepsis has yet been published. The present study was designed to determine whether lower volume of resuscitation fluid and a shorter time to HDS could be achieved in patients with severe sepsis treated with 6% HES 130/0.4 vs. a control group treated with crystalloid (NaCl 0.9%). Another objective of the present trial was to assess the occurrence of potential adverse effects, such as kidney dysfunction, coagulation disorders, and pruritus.

## Materials and methods

### Study design

This prospective, multicentre, active-controlled, double-blind, randomized, clinical study conducted in France and Germany enrolled patients suffering from severe sepsis. Patients received either 6% HES 130/0.4 (colloid treatment group) or sodium chloride (NaCl 0.9%) (crystalloid control group), hereafter referred to as the HES and NaCl groups. The maximum allowed dose for both treatment groups was 50 ml/kg/day (≤8 × 500 ml bags/day for patients weighing ≥80 kg) on the first day and 25 ml/kg/day (≤4 × 500 ml bags/day for patients weighing ≥80 kg) from the second to the fourth day. If extra fluid was required beyond this daily volume and four-day time period, fluid resuscitation was to be carried out using intravenously administered crystalloids (with no volume limitation).

**Figure 1:**
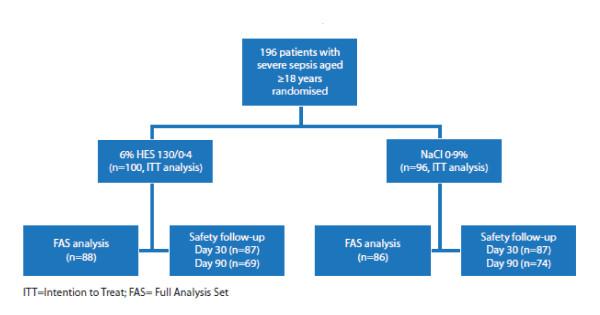
**Study design**.

The investigational and control drugs were identical in appearance and packaging, and were labeled with randomization numbers (20 bags per randomization number) using a blinding methodology as previously described [9]. In order to ensure sufficient hydration, additional crystalloid infusions were requested and given in a ratio to study medication of 1:2. The patient flow of the study is summarized in Figure 1.

### Patients

Patients aged ≥18 years, who required fluid resuscitation, and who had clinically defined severe sepsis, were included in the study [3]. The main exclusion criteria are summarized in Table 1.

**Table 1:** Exclusion criteria.

Related to pre-existing renal impairment
- Known serum creatinine >3.39 mg/dl^a^
- Anuria lasting more than 8 hours despite fluid resuscitation
- Requirement for renal support (either continuous or discontinuous techniques, including intermittent hemodialysis, hemofiltration, and hemodiafiltration)

**Related to the potential effect on the primary endpoint**

- Volume expansion with >3 L of fluid (crystalloid and/or colloid) since diagnosis of severe sepsis or refractory septic shock^b^
- Patients receiving norepinephrine or epinephrine at a dose >0.5 μg/kg/min or dopamine at a dose >15 μg/kg/min at the time of screening

^a^If a creatinine value >3.39 mg/dl became available only after beginning treatment with the study drug, treatment could be continued if the risk/benefit ratio for the individual patient was regarded as positive by the investigator. ^b^Patients treated with low dose vasopressors were not excluded provided they were responsive to fluid resuscitation as demonstrated by an individual fluid challenge.

### Regulatory, ethical and safety issues

The protocol and subsequent protocol amendments were approved by the French Independent Ethics Committee (IEC), the German IEC, and the Competent Authorities. This study was conducted to fulfill a post-marketing requirement of HES 130/0.4 (Voluven^® ^) requested by the US Food and Drug Administration (FDA). Direct written consent from individual patients or legally acceptable representatives was obtained, as well as deferred written informed consent according to country-specific legal requirements.

A data safety monitoring board consisting of two independent clinicians and one independent statistician was established before study commencement to regularly review de-blinded data.

### Assessments

The primary endpoint was the amount of study drug (ml) required to achieve initial HDS, defined as a mean arterial pressure (MAP) ≥65 mmHg and at least two of the following three parameters maintained for four hours: central venous pressure (CVP) between 8 and 12 mmHg, urine output >2 ml/kg, and central venous oxygen saturation (ScvO_2_) ≥70%. In addition, no increase in the infusion of vasopressors or inotropic therapy, and only additional study drug administration of ≤1 L were allowed within these four hours; initial HDS was considered to be achieved at the end of this four-hour period.

The secondary objectives of this study were to explore the efficacy of HES vs. NaCl regarding time taken to achieve initial HDS, total quantity of study drug infused over four consecutive days in the intensive care unit (ICU), length of stay (LOS) in the ICU and in hospital and area under the curve (AUC) of Sequential Organ Failure Assessment (SOFA) score from screening to day 4.

Safety variables were kidney function categorized according to the Risk, Injury, Failure, Loss, End-Stage Kidney Disease (RIFLE) [10] and Acute Kidney Injury Network (AKIN) classifications [11]. Acute renal failure (ARF) was additionally assessed as previously described [5]. Urinary biomarkers of acute kidney injury (AKI), namely Beta-N-acetyl-beta-D-glucosaminidase (NAG), neutrophil gelatinase-associated lipocalin (NGAL) and alpha-1 microglobulin [12], were assessed in a single central laboratory during the first eight days. Laboratory parameters and blood gas analysis were measured at screening and then once daily until the day after last administration of the study drug.

### Statistical analysis

A sample size of 86 patients per group was required to detect a difference of 400 ml of study medication between the group means (90% power), with common standard deviations of 800 ml at a significance level (alpha) of 0.025 using a one-sided *t*-test. In total, 180 patients (90 per group) were randomized according to the original protocol. The Full Analysis Set (FAS) of patients was the primary population for statistical analysis of efficacy and was defined as all randomized patients treated with study drug who reached HDS. All randomized patients, the intention-to-treat (ITT) population, were analyzed for safety variables. The amount of study drug required to achieve initial HDS (primary efficacy endpoint) was tested using a one-sided *t*-test with a type I error ≤2.5%. Pearson's χ^2 ^test was conducted to investigate the frequency of patients not reaching HDS within 48 hours and to analyze mortality rates, renal dysfunction at study inclusion, ARF, and oliguria. *T*-tests were used to further examine the primary efficacy endpoint, volume of red blood cell (RBC) transfusions, and biomarkers of AKI. A chi square (χ^2^)-test was used to test the non-inferiority of HES to NaCl regarding risk of ARF. Kolmogorov-Smirnov tests were carried out to test for normality of biomarker values. Cochran-Mantel-Haenszel tests for trend were used to examine AKIN and RIFLE classifications. *F*-tests of equal variances were carried out to determine whether *t*-tests assuming equal or unequal variances were to be applied. A forward selection logistic regression was conducted to identify independent variables with an effect on mortality and test for a treatment effect (χ^2^-test) after adjusting for these independent variables. The statistical analysis plan was finalized and accepted by the US Food and Drug Administration (FDA) prior to unblinding and analysis of the data.

## Results

Patients were randomized in 24 centers (171 patients from 21 centers in France and 25 patients from three centers in Germany). One hundred patients were randomized to HES and 96 patients were randomized to NaCl.

### Baseline characteristics

There were no significant differences between treatment groups in demographic and baseline characteristics (Table 2). Use of concomitant medications was comparable between treatment groups. Antibiotics were prescribed for 100 patients (100%) and 94 patients (98%) in the HES and NaCl groups, respectively. Most patients received catecholamines, and fluid intake prior to randomization was 35.5 ± 25.3 ml/kg in the HES group and 39.9 ± 28.6 ml/kg in the NaCl group. There were no clinically significant differences between treatment groups for any vital signs or hemodynamic parameters. Severity of disease, assessed by the Simplified Acute Physiology Score II (SAPS II), was similar for both groups. There was no clinically significant difference between groups in the number of patients with renal impairment at the time of screening (62 [63.9%] and 65 [68.4%] in the HES and NaCl groups, respectively, *P *= 0.51).

**Table 2:** Patient demographic and baseline characteristics.

	HES 130/0.4 (n = 100)	NaCl 0.9% (n = 96)
Gender, n (%)		
- Male	64 (64)	57 59)
- Female	36 (36)	39 (41)
Age, years, mean ± SD	65.8 ± 15.4	65.9 ± 14.7
Race, n (%)		
- Caucasian	96 (96)	93 97)
- Asian	1 (1)	1 (1)
- Black	1 (1)	1 (1)
- Other	2 (2)	1 (1)
Mean body mass index, kg/m2	26.2	26.0
Type of patient, n (%)		
- Medical	73 (73)	70 73)
- Surgical	27 (27)	26 (27)
Renal impairment prior to screening*, n (%)	62 (63.9)	65 (68.4)
SAPS II prior to randomization, mean	50	53
SOFA at screening, mean	7.9	9.1
Fluid input prior to randomization, ml/kg body weight, mean ± SD	35.5 ± 25.3	39.9 ± 28.6
Origin of sepsis, n (%)		
- Lungs	53 (53)	58 (60)
- Abdomen	24 (24)	18 (19)
- Urogenital	8 (8)	14 (15)
- Skin, bone and soft tissue	6 (6)	4 (4)
- Other	5 (5)	2 (2)
- Unknown	4 (4)	2 (2)
- Neurological system	3 (3)	2 (2)
- Ear nose and throat	2 (2)	0 (0)
Causative organism, n (%)		
- Gram-negative bacteria	35 (35)	41 (43)
- Gram-positive bacteria	25 (25)	27 (28)
- Other classes	40 (40)	32 (33)

None of the differences were statistically significant; *known serum creatinine >3.39 mg/dl; SD, standard deviation; SAPS II, simplified acute physiology score; SOFA, sequential organ failure assessment; HES, hydroxyethyl starch; NaCI, sodium chloride.

### Efficacy outcomes

The number of patients not reaching HDS within 48 hours after randomization was similar in both groups (12 and 10 in the HES and NaCl groups, respectively, *P *= 0.36). The efficacy analysis included 174 patients who reached HDS (88 patients in the HES group and 86 in the NaCl group; FAS population). Eighty-one patients (81%) in the HES group and 83 patients (87%) in the NaCl group completed the treatment period of four days.

Significantly less HES vs. NaCl was used to reach HDS (1,379 ± 886 ml in the HES group and 1,709 ± 1,164 ml in the NaCl group [Mean difference = -331 ± 1,033, 95% CI -640 to -21], *P *= 0.0185) (Table 3). The median difference was 500 mL in favor of HES, and the maximum dose for initial resuscitation in both groups was 5000 mL. The cumulative volume of study drug used over four consecutive days in the ICU was similar for both groups (2,615 ± 1,499 and 2,788 ± 1,799 for the HES and NaCl groups, respectively). Mean fluid balance from start of study drug to 96 hours thereafter was 56.5 mL/kg body weight in the HES 130/0.4 group and 55.8 mL/kg body weight in the NaCl group. The time to reach HDS was 2.5 hours shorter for the HES group (11.8 ± 10.1 hours vs. 14.3 ± 11.1 hours for NaCl), but the difference was not statistically significant (Table 3).

**Table 3:** Efficacy outcomes.

	HES 130/0.4 (n = 88)	NaCl 0.9% (n = 86)	p
Mean volume of study drug used, ml (SD)	1,379 (886)	1,709 (1,164)	0.0185
Mean time to initial HDS, hours (SD)	11.8 (10.1)	14.3 (11.1)	NS
Number of patients prescribed intravenous catecholamines (%)	88 (88.0)	87 (90.6)	NS

HDS, hemodynamic stabilization; SD, standard deviation; HES, hydroxyethyl starch; NaCI, sodium chloride; NS, not significant.

There was no statistically significant difference between HES and NaCl for LOS in the ICU (15.4 ± 11.1 days vs. 20.2 ± 22.2 days for HES and NaCl, respectively) or LOS in the hospital (37.7 ± 26.5 days vs. 42.7 ± 31.6 days for HES and NaCl, respectively) for patients who did not die before the study ended.

Mean total SOFA score decreased from baseline and was similar at the last available value (5.8 and 6.0 for HES and NaCl, respectively). The AUC of the SOFA score per day, from screening to day 4, was similar for both groups (6.9 ± 3.3 vs. 7.6 ± 3.1 for HES and NaCl, respectively). There were no clinically significant differences between treatment groups for any vital signs or hemodynamic parameters.

Mortality rate until day 28 was 31/100 (31.0%) vs. 24/95 (25.3%) for patients treated with HES or NaCl, respectively (*P *= 0.37). Likewise, mortality rate up to the end of the follow-up period (day 90) was similar between treatments, with no treatment effect on mortality rate detected (40/99 [40%] vs. 32/95 [34%]) for HES and NaCl, respectively (*P *= 0.33). After adjusting for SAPS II, the treatment had no statistically significant effect on mortality until the end of the follow-up period (*P *= 0.1721). A forward selection logistic regression analysis identified SAPS II, site of sepsis, causative organism and age to be associated with mortality, while fluid treatment had no significant effect (*P *= 0.3754).

### Safety outcomes

Safety outcomes were assessed for the ITT patient population (196 patients) (Figure 1).

**Figure 2:**
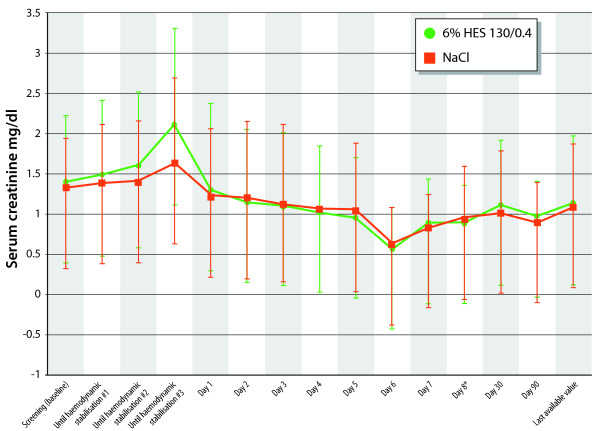
**Evolution of mean serum creatinine (SCr) levels over time**.

Treatment groups were comparable for the RIFLE and AKIN classifications (*P *= 0.81 and 0.59, respectively) and for the number of patients presenting with ARF at any time after screening (24 [24.5%] vs. 19 [20%] for HES and NaCl, respectively, *P *= 0.454; Table 4). The course of mean serum creatine (SCr) over time was also similar in both groups (Figure 2). The mean highest SCr values were 1.757 ± 1.230 and 1.722 ± 1.195 mg/dl (155 ± 109 and 152 ± 106 µmol/l, respectively) for HES and NaCl, respectively (*P *= 0.93). Urinary biomarkers indicated that HES did not induce AKI (Table 5), with neither tubular nor glomerular function significantly affected. Differences between treatment groups were not significantly different for coagulation factors. In addition, there were no significant differences between HES and NaCl for number of patients receiving RBC transfusion or volumes of RBC transfusions (Table 6). Overall, three patients in the HES group (3.0%) and three patients in the NaCl group (3.1%) experienced itching. Eleven patients (11%) in the HES group and 16 patients (16.7%) in the NaCl group had nosocomial infections. At no time, did the Data & Safety Monitoring Board recommend the study to be put on hold or enrolment to be discontinued due to safety issues related to the study medication.

**Table 4:** Number of patients by the AKIN and RIFLE classifications

Worst AKIN stage	HES 130/0.4 (n = 100)na (nb) (%)c	NaCl 0.9% (n = 96)n^a ^(n^b^) (%)^c^
None	100 (52) (52.0)	96 (52) (54.2)

AKIN Stage 1	100 (21) (21.0)	96 (21) (21.9)

AKIN Stage 2	100 (5) (5.0)	96 (6) (6.3)

AKIN Stage 3	100 (22) (22.0)	96 (17) (17.7)

*P *value of test for trend	0.5857	

**Worst RIFLE component**		

None	100 (77) (77.0)	96 (73) (76.0)

**R**isk	100 (13) (13.0)	96 (11) (11.5)

**I**njury	100 (4) (4.0)	96 (5) (5.2)

**F**ailure	100 (5) (5.0)	96 (7) (7.3)

**L**oss	100 1 (1.0)	96 (0) (0.0)

**E**nd-stage kidney disease	100 0 (0.0)	96 (0) (0.0)

*P *value of test for trend	0.8082	

The AKIN classification was based on serum creatinine values and renal replacement therapy.. Urine output criteria were ignored. The RIFLE classification was based on serum creatinine values. Urine output criteria were ignored. ^a^Number of evaluable patients (those for whom a score could be determined); ^b^number of patients analysed; ^c^percentages based on the number of evaluable patients; AKIN, Acute Kidney Injury Network; RIFLE, risk, injury, failure, loss, end-stage kidney disease; HES, hydroxyethyl starch; NaCI, sodium chloride.

**Table 5:** Mean urinary biomarkers of acute kidney injury as a ratio to urinary creatinine.

	Mean (SD)							
**Treatment group**	**Baseline**	**Until HDSa**	**Day 1**	**Day 2**	**Day 3**	**Day 4**	**Day 8b**	**Lastc**

**Alpha-1-microglobulin/urinary creatinine, g/mmol**								

HES 130/0.4	17.8 (21.0)	18.1 (14.8)	18.3 (16.0)	19.4 (20.3)	19.6 (20.5)	17.2 (14.4)	13.4 (14.9)	19.9 (22.7)
NaCI 0.9%	12.3 (12.9)	17.2 (18.0)	17.8 (17.1)	16.9 (15.0)	16.7 (14.9)	16.7 (13.8)	19.5 (23.9)	19.8 (23.3)

**Beta-NAG/urinary creatinine, UI/mmol**								

HES 130/0.4	4.9 (6.6)	4.1 (3.5)	5.0 (3.8)	7.9 (12.8)	8.1 (13.6)	5.5 (4.6)	4.5 (3.0)	6.7 (10.0)
NaCI 0.9%	4.1 (4.7)	4.2 (3.5)	6.0 (9.1)	4.7 (4.2)	4.5 (4.4)	4.2 (3.3)	5.8 (5.0)	5.7 (5.5)

**NGAL/urinary creatinine, μg/mmol**								

HES 130/0.4	283.0 (785.1)	352.8 (710.7)	229.9 (465.5)	325.9 (1,079.0)	432.9 (1458.2)	90.9 (203.4)	24.4 (71.5)	279.0 (884.8)
NaCI 0.9%	305.5 (833.9)	244.9 (452.4)	318.7 (644.8)	149.8 (303.2)	121.1 (306.1)	112.1 (373.7)	177.8 (551.5)	212.8 (604.8)

^a^First measurement until HDS visit (i.e., data recorded at day of withdrawal were assigned to the study visit corresponding to the actual time point of measurement); ^b^data recorded on Day 8; ^c^last available post-baseline measurement; HES, hydroxyethyl starch; NaCI, sodium chloride; NAG, N-acetyl-β-D-glucosaminidase; NGAL, neutrophil gelatinase associated lipocalin; SD, standard deviation; HDS, hemodynamic stabilization.

**Table 6:** Bleeding and coagulation.

	HES 130/0.4(n = 100)		NaCl 0.9%(n = 96)		*P *value
Number of patients with RBC transfusions, n (%)	29 (29.0)		20 (20.8)		0.2480
Volume (ml) of RBC transfusions, n (mean ± SD, range)	100 (214 ± 358, 0 to 1,394)		96 (165 ± 354, 0 to 1,661)		0.3415
Blood loss, ml, including blood sampling and drainage, n (mean ± SD, range)	63 (10.0 ± 24.4, 0.4 to 150.7)		65 (10.5 ± 29.4, 0.2 to 207.8)		NS

**Coagulation parameters, median values over time**	**INR**	**aPTT (ratio)**	**INR**	**aPTT (ratio)**	***P *value**

Baseline	1.3	1.2	1.3	1.2	
Until HDS^a^	0.1	0.2	0.0	0.1	
Day 1	0.0	0.1	0.0	0.1	
Day 2	-0.1	0.1	-0.1	0.0	
Day 3	-0.1	0.1	-0.2	0.0	
Day 4	-0.2	0.1	-0.2	-0.1	
Day 5	-0.1	0.0	-0.1	-0.1	
Day 8^b^	-0.1	0.0	-0.2	0.0	
Last^c^	-0.1	0.0	-0.2	0.0	

^a^First measurement until HDS visit (i.e., data recorded at day of withdrawal were assigned to the study visit corresponding to the actual time point of measurement); ^b^data recorded on Day 8; ^c^last available post-baseline measurement; RBC, red blood cell; SD, standard deviation; HDS, hemodynamic stabilization; HES, hydroxyethyl starch; NaCI, sodium chloride.

## Discussion

This is the first randomized, controlled, prospective, multicentre, double-blind study of 6% HES 130/0.4 in patients suffering from severe sepsis. The significantly lower volume to achieve initial HDS vs. NaCl confirms the good volume expansion effect of 6% HES130/0.4 [13]. The safety profile of HES is an ongoing matter of debate. Several factors should be considered when assessing potential drawbacks of HES: the type of HES (concentration, molar substitution, molecular weight, and C_2_/C_6 _ratio) and daily and cumulative doses used, underlying baseline kidney dysfunction, the case mix (surgery, trauma, or sepsis), and ensuring patients are sufficiently hydrated.

There have been initial concerns regarding the negative effect of older HES products on kidney function after renal transplant [14]. More recent studies with third generation HES have not confirmed this effect on kidney function [15,16]. For patients with sepsis receiving older HES products for fluid resuscitation, two randomized studies have documented an alteration of renal function [5,6]. In the first study, a randomized, multicentre study including 129 septic shock patients, patients resuscitated with 6% HES 200/0.62 had a higher incidence of ARF vs. patients treated with gelatin (42% vs. 23%, *P *< 0.03) [5]. The volumes received and the levels of baseline creatinine before vascular filling in the two treatment arms, however, were different, thus rendering a head-to-head comparison difficult. Nonetheless, multivariate analysis showed that using 6% HES 200/0.62 was an independent risk factor for secondary formation of renal insufficiency. In the second study, a multicenter, two-by-two factorial trial including 537 patients with severe sepsis, resuscitation with a hyperoncotic 10% HES 200/0.5 given at very high cumulative volumes, was associated with higher rates of ARF and RRT vs. Ringer's lactate [6]. Basic hydration therapy has been criticized as being insufficient in both these trials and in the second study almost 80% of patients were randomized after successful initial HDS. Whether or not the association between ARF and the liberal infusion of the hyperoncotic HES preparation was cause or effect remains elusive.

In the current study, the number of patients presenting with ARF, defined as doubling of the baseline creatinine value or need for RRT, was similar for 6% HES 130/0.4 and NaCl 0.9% (24.5% vs. 20%, respectively). Treatment groups were also comparable for the AKIN and RIFLE classifications. Likewise, results from three urinary biomarker analyses confirmed that 6% HES 130/0.4 did not induce AKI, because neither the tubular, nor the glomerular function was affected. In addition, there was no statistically significant treatment difference in median change in SCr from baseline or peak post-baseline SCr. These findings are in agreement with a recent observational study in patients with severe sepsis or septic shock, which did not find an association of HES 130/0.4 with RRT or renal dysfunction [17].

The fact that 6% HES 130/0.4 has a good renal safety profile [18-22] was recently confirmed in a randomized trauma study [23]. Even though very high volumes of HES 130/0.4 (approximately 70ml/kg) were infused during initial resuscitation, the incidence of renal dysfunction was lower in the HES group vs. patients treated with NaCl 0.9%. Patients with severe underlying kidney failure were excluded from the present study. However, importantly, in a pharmacokinetic study in which 500 ml 6% HES 130/0.4 was administered to each of 19 patients with pre-existing renal insufficiency of variable degree, the maximum plasma concentration of starch and its terminal half-life were not affected by renal insufficiency [24].

Besides the study drug, patients enrolled in the present study received basic daily rehydration therapy with 500 ml of infused isotonic saline per two bags of study drug. The cumulative volume of study drug was restricted to a maximum of 4,000 ml on day 1 (50 ml/kg) and 2,000 ml/day for days 2 to 4 (10 L in total). As a consequence, the mean cumulative volume of 2,500 to 3,000 ml was much lower, and the duration of 6% HES 130/0.4 administration much shorter (4 days) compared to the 'prospective randomized multicenter study on the influence of colloid vs. crystalloid volume resuscitation and of intensive vs. conventional insulin therapy on outcome in patients with severe sepsis and septic shock' (VISEP) trial, where 38% of patients repeatedly received more than the allowed maximum daily dose for a second generation starch and duration of administration was >3 weeks for 13% of the patients [6].

Apart from concerns regarding effects on kidney function, effects of different HES types on coagulation and bleeding events have been investigated in depth. In this regard, it is important to note that the influence on coagulation of 6% HES 130/0.4 is significantly reduced vs. older HES types. Unlike HES types with a high molar substitution, trials with 6% HES 130/0.4 in doses of up to 50 ml/kg body weight in cardiac surgery [22], abdominal surgery [25], and up to 70 ml/kg bodyweight in severe cranio-cerebral trauma [21] have revealed no deterioration in coagulation. The decreased influence on coagulation and hence decreased incidence of undesirable effects after 6% HES 130/0.4 vs. older products is reflected in decreased blood loss in surgical patients, and subsequent transfusion requirements [26]. The results of the present study are in harmony with the latter trials and confirm that 6% HES 130/0.4 has no clinically relevant impact on coagulation when used correctly. In the VISEP trial, the component of the SOFA score related to coagulation was more altered in the HES group vs. crystalloids [6]. Conversely, we did not find any differences in blood loss or RBC transfusion requirements. There was no significant difference between treatment groups for any laboratory coagulation parameters.

Mortality rates were not statistically different between treatment groups. An important finding was a difference in initial mortality rate in the 6% HES 130/0.4 group, possibly due to a higher rate of patients with abdominal sepsis. This difference continued until day 90. Interestingly, the pattern of mortality rates was different from that observed in the VISEP study [6], where a non-significant separation started after day 30. The type of volume resuscitation used was not an independent risk factor for mortality in our study.

Other side effects were similar between treatment groups. In animal experiments in rats, 6% HES 130/0.4 has been shown to significantly reduce tissue storage (75% lower) for the whole body compared with HES 200/0.5 [27]. Deposits of HES in skin have been linked to the occurrence of pruritus [28]. Three patients in each group experienced itching in the current study and our results clearly demonstrate that tetrastarch infusion as used in the present study was not linked to itching.

This study has some limitations. First, no algorithm to assess fluid responsiveness was used and CVP, though still recommended by the surviving sepsis guidelines, is known to be a poor indicator of preloading [29]. Second, the study was not designed or powered to assess effects on mortality. A previous study has shown a tendency for reduced mortality in patients with severe sepsis if resuscitated with colloids vs. crystalloids [30]. The 'crystalloid vs. hydroxyl-ethyl starch trials' (CHEST) study, which is using 6% HES 130/0.4, will probably settle this debate [9]. Finally, though extensive biomarker analyses are provided and objective criteria to assess kidney function were used, effects on kidney function were not powered to assess an effect. Besides the CHEST study, another ongoing larger study [31] will address this important issue.

## Conclusion

The primary goal of the study was reached since HES 130/0.4 was found significantly superior to NaCl with respect to the amount of study drug required to achieve initial hemodynamic stability in patients suffering from severe sepsis. As used in our study, 6% HES 130/0.4, had no negative effects on mortality, kidney function, coagulation, or pruritus. These results need to be confirmed by larger trials.

## Key messages

• Significantly less HES was used to reach HDS vs. NaCl (1,379 ± 886 ml in the HES group and 1,709 ± 1,164 ml in the NaCl group [mean difference = -331 ± 1,033, 95% CI -640 to -21], *P *= 0.0185)

• Mortality rate until day 28 was 31/100 (31.0%) vs. 24/95 (25.3%) for patients treated with HES or NaCl, respectively (*P *= 0.37). Likewise, mortality rate up to the end of the follow up period (day 90) was similar between treatments, with no treatment effect on mortality rate detected (40/99 [40%] vs. 32/95 [34%]) for HES and NaCl, respectively (*P *= 0.33).

• Treatment groups were comparable for the RIFLE and AKIN classifications (*P *= 0.81, and 0.59, respectively) and for the number of patients presenting with ARF at any time after screening (24 [24.5%] vs. 19 [20%] for HES and NaCl, respectively, *P *= 0.454).

• Overall, three patients in the HES group (3.0%) and three patients in the NaCl group (3.1%) experienced itching.

## Abbreviations

AKI: acute kidney injury; AKIN: acute kidney injury network; ARF: acute renal failure; AUC: area under the curve; CVP: central venous pressure; FAS: full analysis set; HDS: hemodynamic stabilization; FDA: US Food and Drug Administration; HES: hydroxyethyl starch; ICU: intensive care unit; LOS: length of stay; MAP: mean arterial pressure; NaCI: sodium chloride; NAG: Beta-N-acetyl-beta-D-glucosaminidase; NGAL: neutrophil gelatinase-associated lipocalin; RBC: red blood cells; RIFLE: risk: injury: failure: loss: end-stage kidney disease; RRT: renal replacement therapy; SAPS II: simplified acute physiology score; Scr: serum creatinine; ScvO_2_: central venous oxygen saturation; SOFA: sequential organ failure assessment.

## Competing interests

Bertrand Guidet has honoraria and financial reimbursements from Fresenius Kabi for lecturing and authorship as well as honoraria from Laboratoire Français du Fractionnement et des biotechnologies for lecturing, and has received an unrestricted educational grant from Fresenius Kabi Deutschand GmbH (study sponsor) for the CRYSTMAS Study. Fresenius Kabi was involved in the study design, analysis and preparation of the report.

## Authors' contributions

BG was responsible for the literature search, design of the study, data collection, data interpretation and writing the manuscript with the help of a medical writer. He takes full responsibility for the manuscript. All other authors contributed to study design, patient enrolment, data collection and validation of the manuscript. All authors read and approved the final manuscript submitted for publication.
